# Role of radiotherapy in the management of malignant airway obstruction

**DOI:** 10.1111/1759-7714.13523

**Published:** 2020-06-12

**Authors:** Hoon Sik Choi, Bae Kwon Jeong, Hojin Jeong, In Bong Ha, Ki Mun Kang

**Affiliations:** ^1^ Radiation Oncology Gyeongsang National University School of Medicine, and Gyeongsang National University Changwon Hospital Changwon‐si South Korea; ^2^ Radiation Oncology Gyeongsang National University School of Medicine, and Gyeongsang National University Hospital Jinju‐si South Korea; ^3^ Institute of Health Science Gyeongsang National University Jinju‐si South Korea

**Keywords:** Lung neoplasm, malignant airway obstruction, palliative, radiotherapy

## Abstract

**Background:**

A significant proportion of lung cancer patients suffer from malignant airway obstruction (MAO). Palliative external beam radiotherapy (EBRT) is often used to control the symptoms caused by MAO. In this study, we report the effect of palliative EBRT on lung cancer with MAO and analyze the factors that influence it.

**Methods:**

This study included 75 patients with MAO in lung cancer who underwent palliative EBRT, between 2009 and 2018 and were analyzed retrospectively. Change of dyspnea, tumor response, and overall survival (OS) were recorded. Univariate and multivariate analyses were performed to determine the prognostic factors for treatment outcomes.

**Results:**

The median follow‐up duration was 2.5 months, and median OS was 2.3 months. Out of 75 patients, dyspnea was improved in 46 patients (61.3%), and tumor was partially decreased in 39 patients (52%). Symptoms improved in all tumor responding patients. The symptom improvement was significantly affected by radiation dose and time to EBRT. The tumor response was significantly affected by pathology, radiation dose, and time to EBRT.

**Conclusions:**

Palliative EBRT is an effective and safe treatment option for patients with MAO in lung cancer. In particular, high‐dose irradiation and prompt treatment can improve treatment results.

**Key points:**

**Significant findings of the study:**

In MAO patients, tumor response is an important factor for resolving dyspnea and improving survival rate. In order to increase the tumor response, high‐dose irradiation and prompt treatment after symptoms occur are necessary.

**What this study adds:**

Our study reported the effects of EBRT and prognostic factors in MAO patients. We emphasize that palliative EBRT is a relatively safe and effective treatment in MAO patients, which is a complement to previous studies.

## Introduction

Lung cancer is the most commonly diagnosed cancer worldwide and the most common cause of cancer‐related death.^1^ Although advances in imaging technology have made it possible to detect early‐stage lung cancer, most lung cancer patients are still diagnosed at an advanced stage.^2^ In this late course of disease, bulky and progressed intrathoracic tumors can cause symptoms such as cough, hemoptysis, chest pain, superior vena cava syndrome, hoarseness, or dyspnea from malignant airway obstruction (MAO).^3^

For MAO, a third of lung cancer patients are obstructed at diagnosis, and a significant proportion of the other patients will develop obstruction at some point in the course of the disease.^4^ MAO can cause pneumonia as well as dyspnea, and can be an immediate cause of death, so if possible, it requires immediate treatment.^5^

Various palliative‐intent treatments are attempted to improve symptoms and penetrate blocked airways.^4^, ^6^ External beam radiotherapy (EBRT) is a preferred treatment option for MAO because it is noninvasive, safe, and simpler than other methods such as a bronchoscopic procedure, laser ablation, and intraluminal brachytherapy.^7^, ^8^ However, there are several limitations in EBRT in that there is no standardized guideline (radiation dose, fractionation, and time for EBRT) and the result data are insufficient.

Therefore, in this study, we report our institutional experience and treatment outcomes of treating lung cancer patients with MAO by palliative EBRT. We analyze how treatment by EBRT is better for symptom relief, good tumor response, and patient survival.

## Methods

### Patient selection

In this study, the inclusion criteria were as follows: (i) Patients with histologically‐proven primary lung cancer; (ii) patients suffering from dyspnea with a radiographic finding of MAO on chest X‐ray or computed tomography (CT) scan; and (iii) patients treated by palliative‐intent EBRT for an obstructive pulmonary mass. Patients who received prior systemic chemotherapy were included in this analysis. In contrast, patients who had any of following conditions were excluded from this study: (i) No follow‐up image data which showed the treatment response; (ii) no follow‐up medical records which showed the change of symptoms; and (iii) a previous history of RT and surgery at chest. Among those patients who received palliative EBRT at Gyeongsang National University Hospital (GNUH) and Gyeongsang National University Changwon Hospital (GNUCH) between November 2009 and December 2018, we selected 75 patients who fully fulfilled the above criteria for analysis and retrospectively reviewed their medical charts, treatment records, and the results of image work‐up.

This study was retrospective, with no informed consent from individual patients, but was done in accordance with the relevant guidelines; the study protocol was approved by the Institutional Review Boards (IRBs) at GNUH (IRB No. GNUH 2020–03‐009) and GNUCH (IRB No. GNUCH 2020–03‐020).

### Radiotherapy

For radiotherapy (RT), all patients were immobilized in the supine position and had previously received CT simulations. The scanned images were imported into the Eclipse treatment‐planning system. Total or partial lung mass including conglomerated metastatic lymph nodes presumed by clinicians to induce airway obstruction was delineated as gross tumor volume. The clinical target volume was not delineated, because treatment was delivered with palliative intent. Subsequently, a volumetric margin of 10 mm was applied to make the planning target volume (PTV). Three‐dimensional conformal RT plans were created and used to prescribe a median dose of 39 Gy (range, 24–59 Gy), 2–3 Gy per fraction, equal to a median equivalent dose in 2 Gy per fraction (EQD2) 42.2 Gy (range, 26–62.2 Gy). All plans were normalized so that 100% of PTV received more than 90% of the prescribed dose Commonly used RT dose prescription protocols are summarized in Table 1.

**Table 1:** Patient characteristics

Variable	No. of patients	(%)
Age	Median 68 years	Range 49–84 years
Sex		
Male	60	80.0
Female	15	20.0
Smoking history		
Yes	66	88.0
No	9	12.0
Comorbid COPD		
Yes	16	21.3
No	59	78.7
ECOG PS		
0–1	38	50.7
2	22	29.3
3	15	20.0
Pathology		
NSCLC	52	69.3
SCLC	23	30.7
Disease status		
Untreated	24	32.0
Relapse or refractory	51	68.0
Degree of obstruction		
Partial	56	74.7
Total	19	25.3
Carina involvement		
Yes	37	49.3
No	38	50.7
Tumor length		
<5.6 cm	31	41.3
≥5.6 cm	44	58.7
ATS score (before RT)		
2	9	12.0
3	32	42.7
4	34	45.3
RT dose regimens		
39 Gy in 13 fractions	25	33.3
36 Gy in 12 fractions	12	16.0
30 Gy in 10 fractions	12	16.0
Others	26	34.7

ATS, American Thoracic Society; COPD, chronic obstructive pulmonary disease; ECOG, Eastern Cooperative Oncology Group; No., number; NSCLC, non‐small cell lung cancer; PS, performance status; RT, radiotherapy; SCLC, small cell lung cancer.

If the changes in atelectasis or airway position were observed by means of daily chest X‐rays during RT, the previous procedure was repeated from CT scanning to RT planning. We then continued with the remaining RT with the new RT plan.

### Statistical analysis

The severity of dyspnea was recorded by radiation oncologists based on the American Thoracic Society (ATS) score, and the differences in score before, during, and after RT was used to assess symptom improvement.^9^ We defined time for EBRT as the period from the day of dyspnea with an ATS score of 2 or higher to the day of RT start. In simulated chest CT images, the degree of obstruction, presence of carina involvement, and length of the tumor blocking the airway were measured. On mean 27 days (range, 4–90 days) after the end of RT, all patients had scanned chest CT for treatment response evaluation. Tumor response was divided into complete response (CR), partial response (PR), progressive disease (PD), and stable disease (SD) according to the Response Evaluation Criteria In Solid Tumors (RECIST) criteria, and we defined CR or PR in the RT field area as the responding group.^10^ Acute toxicity was evaluated by National Cancer Institute's Common Terminology Criteria for Adverse Events (CTCAE) version 4.0.^11^

Simple and multivariate logistic regression analyses were done to identify prognostic factors of symptom relief and tumor response. The overall survival duration was defined as the period from the date of end of RT to the date of any death. Kaplan‐Meier method and Log‐rank test were used for survival curves. All analyses were done using the SPSS program, and a *P*‐value <0.05 was considered statistically significant.

## Results

### Patient characteristics

Patient characteristics are summarized in Table 1. There was a total of 75 patients (GNUH, 48 patients; GNUCH, 27 patients) enrolled into this study, their median age was 68 (range 49–84 years) and most were male (80%). Their Eastern Cooperative Oncology Group (ECOG) performance scores were 0 (1 patient), 1 (37 patients), 2 (22 patients), and 3 (15 patients). The histology was small cell lung cancer (SCLC) in 23 (30.7%) patients and non‐small cell lung cancer (NSCLC) in 52 (69.3%) patients: squamous cell carcinoma, 37 patients; and adenocarcinoma, 15 patients. At the time of complaint of dyspnea, 51 (68%) patients had lung cancer with relapse or refractory disease status after palliative chemotherapy; the other 24 (32%) patients had untreated, just‐diagnosed lung cancer. Most of the patients (92.5%) did not receive chemotherapy during the course of RT. On initial chest CT, the degree of obstruction was partial in 56 (74.7%) patients and total in 19 (25.3%). The number of carina‐involved patients was 37 (49.3%) out of 75 patients. All of the patients with total obstruction had carina involvement. The median length of the tumor blocking the airway was 5.6 cm (range, 3.2–9.7 cm). The dyspnea level of patients before RT was divided into two, three, and four points based on ATS score, for nine, 32, and 34 patients, respectively. No patient had a bronchial stent prior to EBRT. Time for EBRT was median 14 days (range, 1–112 days).

### Treatment outcomes

**Figure 1:**
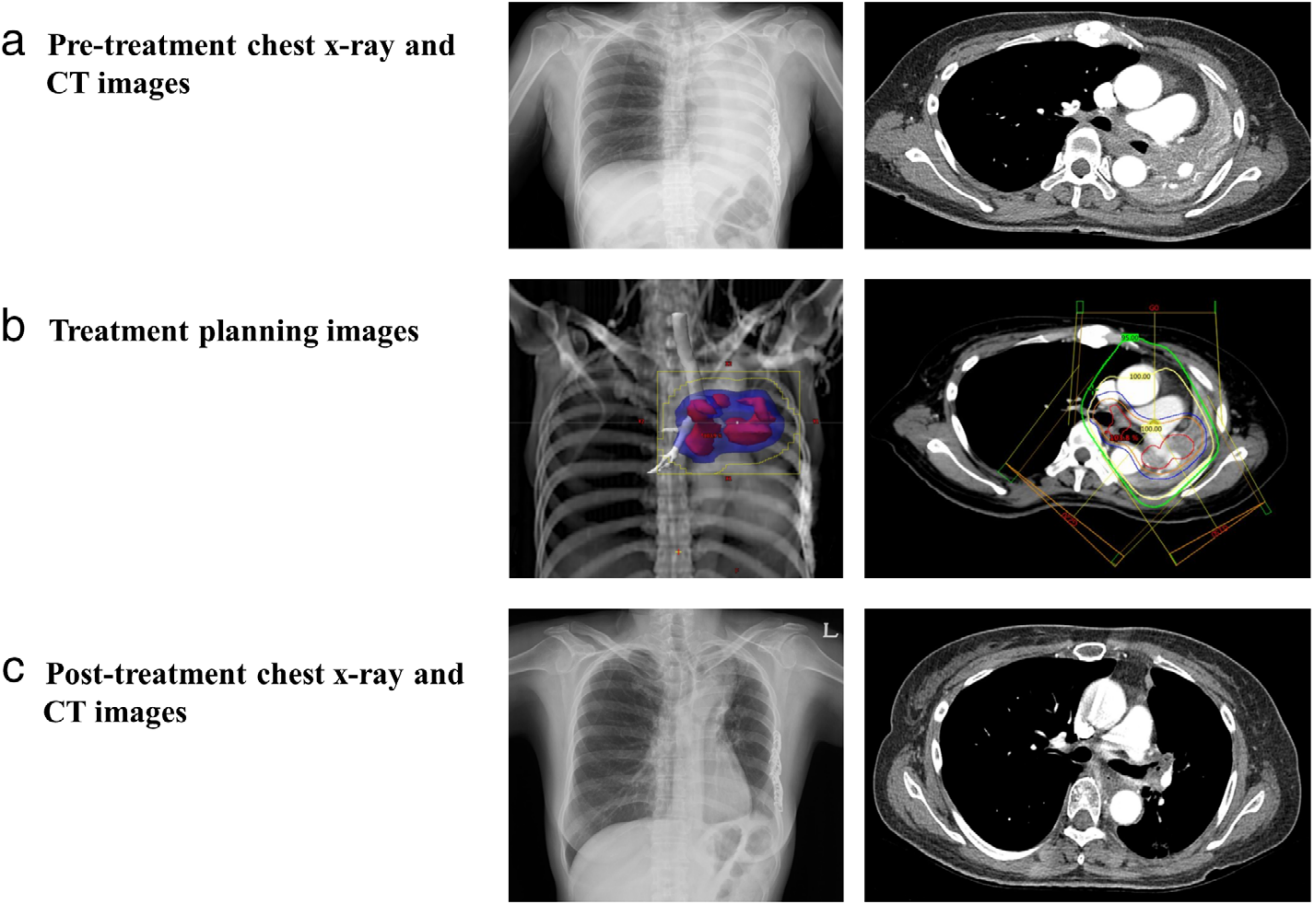
Images for a patient who underwent palliative radiotherapy for malignant airway obstruction. **a** Pretreatment chest X‐ray and CT images; **b** Treatment planning images; and **c** Post‐treatment chest X‐ray and CT images.

The median follow‐up duration was 3.4 months (range, 0.2–32.2 months), and 71 patients (94.7%) had died at the end of the follow‐up period. The median overall survival (OS) was 3.4 months, and one‐year OS rate was 15.9%. The degree of symptomatic change in dyspnea was divided based on the difference in ATS scores before and after RT, as shown in Table 2. According to the ATS score gap, the number of patients with 3, 2, and 1 was eight (10.7%), 18 (24%), and 20 (26.7%), respectively. In 46 patients (61.3%), the symptoms of dyspnea improved, although there was a difference in degree. On the other hand, 24 patients (32%) had no change in symptoms, and five patients (6.7%) symptoms had worsened despite treatment. Tumor response was classified into CR, PR, SD, and PD and the number of patients was 0 (0%), 39 (52%), 29 (36%), and seven (9.3%), respectively. A total of 52% of patients showed a tumor response after RT. All patients with no improvement in their symptoms had no tumor response, and all patients with tumor responses showed symptom improvement. The tumor response in one patient who underwent palliative EBRT is shown in Fig 1 by comparing chest CT images before and after treatment. Based on CTCAE criteria, acute toxicity was grade 1 esophagitis in 21 patients, grade 2 esophagitis in 10 patients, and grade 1 radiation dermatitis in three patients. No patient suffered from grade 3 or higher toxicity.

**Table 2:** Symptom changes before and after radiotherapy based on American thoracic society score

ATS score gap	No. of patients (%)	Symptom change
3	8 (10.7)	Symptom improvement (61.3%)
2	18 (24)	
1	20 (26.7)	
0	24 (32)	No change
−1 or − 2	5 (6.7)	Symptom aggravation

ATS, American Thoracic Society; No., number.

### Prognostic factors

**Figure 2:**
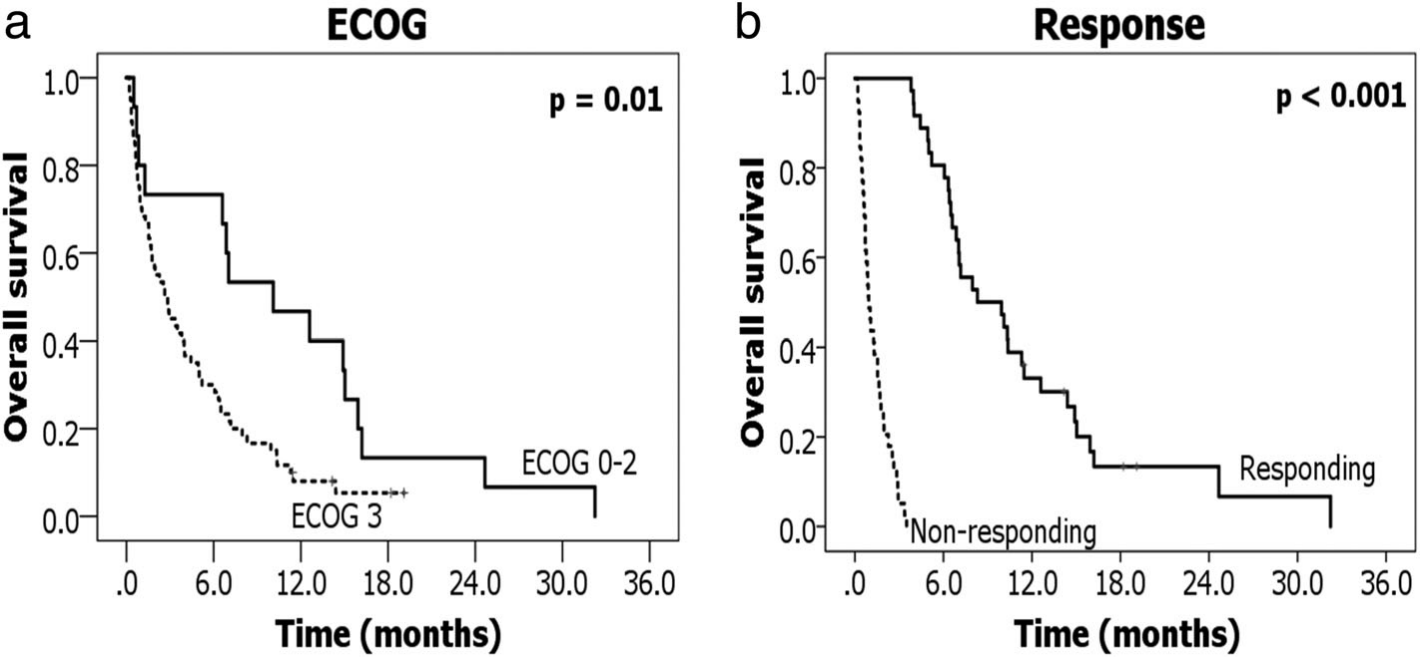
Overall survival according to **a** performance status; and **b** tumor response.

We used logistic regression analyses to find factors that could affect the treatment outcomes. In the univariate analysis, ECOG performance status (*P* = 0.004), disease status (*P* = 0.034), degree of obstruction (*P* = 0.051), tumor length (*P* = 0.001), EQD2 (*P* = 0.008), and time to RT (*P* = 0.014) were significant factors in symptom improvement. EQD2 (*P* = 0.012) and time to RT (*P* = 0.007) remained significant in the multivariate analysis. For tumor response, ECOG performance status (*P* = 0.035), pathology (*P* = 0.004), disease status (*P* = 0.028), degree of obstruction (*P* = 0.044), EQD2 (*P* = 0.005), and time to RT (*P* = 0.001) were statistically significant in univariate analysis. However, pathology (*P* = 0.002), EQD2 (*P* = 0.002), and time to RT (*P* < 0.001) remained significant in the multivariate analysis. The results for symptom improvement and tumor response are shown in Tables 3 and 4. The analysis results for the factors affecting the OS are shown in Fig 2. Patients with an ECOG performance status of 0–2 had a one‐year OS rate of 46.7% and median OS of 10.1 months, whereas those with ECOG performance status 3 had a median OS of 2.6 months; this difference was statistically significant. For tumor response, one‐year OS rate that was statistically significant differed by 33.1% in the responding group and 0% in the nonresponding group.

**Table 3:** Prognostic factors for symptom improvement

Univariate analysis			
Variable	Hazard ratio	95% CI	*P*‐value
Medical center (GNUH vs. GNUCH)	0.873	0.333–2.290	0.782
Sex (Male vs. Female)	0.662	0.211–2.073	0.479
Age (<68 vs. ≥68 years)	1.029	0.943–1.124	0.518
Smoking (No vs. Yes)	1.276	0.503–3.237	0.609
COPD (No vs. Yes)	1.509	0.465–4.899	0.494
Pathology (NSCLC vs. SCLC)	3.086	0.996–9.561	0.051
Dyspnea level before RT (2–3 vs. 4)	1.084	0.548–2.144	0.816
Carina involvement (No vs. Yes)	0.543	0.212–1.392	0.204
ECOG PS (0–2 vs. 3)	0.156	0.044–0.555	**0.004**
DS status (untreated vs. relapse or refractory)	0.296	0.096–0.915	**0.034**
Degree of obstruction (partial vs. total)	0.344	0.118–1.004	**0.051**
Tumor length (<5.6 vs. ≥5.6 cm)	0.160	0.052–0.494	**0.001**
EQD2 (<42.2 vs. ≥42.2 Gy)	3.791	1.411–10.188	**0.008**
Time to RT (≤14 vs. >14 days)	0.289	0.108–0.774	**0.014**
Multivariate analysis			
Variable	Hazard ratio	95% CI	*P* value
EQD2 (<42.2 vs. ≥42.2 Gy)	5.704	1.457–22.340	**0.012**
Time to RT (≤14 vs. >14 days)	0.141	0.034–0.587	**0.007**

Significant values are shown in bold.

CI, confidence interval; COPD, chronic obstructive pulmonary disease; DS, disease; ECOS, Eastern Cooperative Oncology Group; EQD2, equivalent dose in 2 Gy per fraction.; GNUCH, Gyeongsang National University Changwon Hospital; GNUH, Gyeongsang National University Hospital; NSCLC, non‐small cell lung cancer; PS, performance status; RT, radiotherapy; SCLC, small cell lung cancer.

**Table 4:** Prognostic factors for tumor response

Univariate analysis			
Variable	Hazard ratio	95% CI	*P*‐value
Medical center (GNUH vs. GNUCH)	0.991	0.386–2.546	0.985
Sex (Male vs. Female)	0.766	0.246–2.380	0.644
Age (<68 years vs. ≥68 years)	1.007	0.925–1.096	0.875
Smoking (No vs. Yes)	0.942	0.380–2.332	0.897
COPD (No vs. Yes)	1.243	0.409–3.778	0.702
Pathology (NSCLC vs. SCLC)	5.314	1.708–16.536	**0.004**
Dyspnea level before RT (2–3 vs. 4)	1.261	0.645–2.464	0.498
Carina involvement (No vs. Yes)	0.767	0.309–1.901	0.567
ECOG PS (0–2 vs. 3)	0.260	0.074–0.910	**0.035**
DS status (untreated vs. relapse or refractory)	0.312	0.110–0.884	**0.028**
Degree of obstruction (partial vs. total)	0.322	0.107–0.970	**0.044**
Tumor length (<5.6 vs. ≥5.6 cm)	0.418	0.162–1.078	0.071
EQD2 (<42.2 vs. ≥42.2 Gy)	4	1.530–10.457	**0.005**
Time to RT (≤14 vs. >14 days)	0.171	0.063–0.463	**0.001**
Multivariate analysis			
Variable	Hazard ratio	95% CI	*P*‐value
Pathology	12.378	2.604–58.844	**0.002**
EQD2 (<42.2 vs. ≥42.2 Gy)	9.860	2.301–42.258	**0.002**
Time to RT (≤14 vs. >14 days)	0.042	0.009–0.207	**<0.001**

Significant values are shown in bold.

CI, confidence interval; COPD, chronic obstructive pulmonary disease; DS, disease; ECOS, Eastern Cooperative Oncology Group; EQD2, equivalent dose in 2 Gy per fraction; GNUCH, Gyeongsang National University Changwon Hospital; GNUH, Gyeongsang National University Hospital; NSCLC, non‐small cell lung cancer; PS, performance status; RT, radiotherapy; SCLC, small cell lung cancer.

## Discussion

In this study, we analyzed the effects of palliative EBRT and its related factors in 75 patients with lung cancer with MAO. Median EQD2 42.2 Gy was irradiated, 61.3% of patients showed improvement of dyspnea, and 52% of patients showed partial tumor response. The tumor response was good in patients with pathology of SCLC, high‐dose irradiation, or short time to RT. Symptom improvement was better in patients with high‐dose irradiation, or short time to RT. Symptoms improved in all patients with tumor response. The OS of all patients was poor, but that was relatively high in patients with good performance status or good tumor response.

MAO is present in the late course of the disease in a large proportion of lung cancer patients. The prognosis of the patients is very poor and life expectancy is short. However, the symptoms, such as dyspnea, cough, or hemoptysis that accompany MAO, deteriorate the quality of life and require palliative treatment. It is difficult to define standard guidelines, because this treatment should be applied in a variety of ways depending on the patient's overall condition as well as on the severity of the patient's symptoms.

Among palliative treatment methods, EBRT has some advantages that can be easily applied. First, palliative EBRT is effective in lung cancer patients with MAO. Lee *et al*.^7^ gave a median 30 Gy EBRT to 95 patients with airway obstruction in lung cancer. They defined responders as patients with improvement in chest X‐rays or symptoms and reported a total response rate of 78.9%. They also reported that a higher response was observed in patient with a biologically effective dose ≥39 Gy or tumor length ≤ 6 cm. Nihei *et al*.^12^ gave 30 Gy in 10 fractions EBRT to 24 patients with airway stenosis in NSCLC. They assessed treatment response by chest images and reported a response rate of 54.2%, which lasted for a median of 116 days, corresponding to about 66% of the patients’ remaining survival. Our study showed a satisfactory therapeutic effect (symptom response, 61.3%; tumor response, 52%) similar to those of these previous studies. In addition, the effects may be improved by controlling factors such as high‐dose irradiation and short time to RT. Second, palliative EBRT is noninvasive and safe in lung cancer patients with MAO. In MAO patients, therapeutic bronchoscopic procedures are often attempted for palliation. Ernst *et al*.^13^ reported procedure‐related toxicity in 554 patients who received therapeutic bronchoscopy at four hospitals. They reported that general anesthesia was needed in 65.3% of patients and adverse events, such as hypoxia, pneumothorax, escalation of care, bleeding, and hypotension, were found in 25% of patients with malignant tumors. Another study reported the side effects of therapeutic bronchoscopy in 15 institutions and 947 MAO patients.^14^ They reported that side effects could differ depending on the institution and skill of the operator, and if side effects occur, more than 50% of patients develop additional severe adverse events, such as permanent disability or death. On the other hand, in previous studies with EBRT and in our study, grade 3 or higher acute toxicity was not observed in any patients. Although chronic toxicity has not been evaluated, late effects are often less concerning because of their short lifespan in most palliative therapy.

However, there are several disadvantages of palliative EBRT. First, it cannot immediately improve symptoms in MAO patients. Lee *et al*.^7^ reported that the median time for resolving the symptom or radiologic findings was seven days after EBRT, and for Nihei *et al*.^12^ it was 24 days. EBRT alone is not effective in patients with acute phases of respiratory distress or requiring dramatic and immediate symptomatic improvement. This shortcoming can be overcome by combining the bronchoscopic procedure with early effects and the EBRT with delayed effects. Combined treatment studies have reported good results in improving symptom‐free survival and progression‐free survival as well as symptom improvement.^15^, ^16^ Second, palliative EBRT has a relatively long duration of treatment. Because of the short survival of patients with MAO, a long time spent on treatment can be a disadvantage. To overcome this shortcoming, hypofractionated RT can be considered. Theoretically, the risk of late complications increases, but considering the short life expectancy, a faster tumor response can be expected. Additional studies are needed to find proper RT schedules that increase response rates and reduce treatment duration while maintaining low side effects.

The limitation of this study was its retrospective and palliative nature, wherein the patient characteristics and outcome data were not well controlled, which could cause selection bias. However, our study is relatively consistent because both institutions have the same medical staff with the same principles.

In conclusion, in patients with lung cancer with MAO, palliative EBRT is effective and safe. High‐dose irradiation (EQD2 ≥ 42.2 Gy) and prompt treatment (time to RT ≤14 days) may improve the response rate. Finding an optimal dose schedule, to reduce treatment duration and increase response while maintaining toxicity, should be investigated in future studies.

## Disclosure

No authors report any conflicts of interest.
